# Effects of *Alphitobius diaperinus* and *Tenebrio molitor* Larvae Digestates on Intestinal Epithelial Barrier and Antioxidant-Related Gene Expression in Differentiated Caco-2 Cells: An In Vitro Study

**DOI:** 10.3390/biology15191690

**Published:** 2026-09-22

**Authors:** Eleni Dalaka, Constantin S. Filintas, Demeter Lorentha S. Gidari, Maria C. Boukouvala, Georgios Theodorou, Nickolas G. Kavallieratos

**Affiliations:** 1 Laboratory of Animal Breeding and Husbandry, Department of Animal Science, Agricultural University of Athens, 75 Iera Odos str., 11855 Athens, Attica, Greece; gtheod@aua.gr; 2 Laboratory of Agricultural Zoology and Entomology, Department of Crop Science, Agricultural University of Athens, 75 Iera Odos str., 11855 Athens, Attica, Greece; kfilintas@aua.gr (C.S.F.); dlgidari@aua.gr (D.L.S.G.); mbouk@aua.gr (M.C.B.)

**Keywords:** lesser mealworm, yellow mealworm, INFOGEST digestion, Caco-2 monolayer, epithelial effects, molecular response

## Abstract

Edible insects have drawn increasing interest from researchers as sustainable sources of protein with potential functional ingredients for feed and food applications. However, the intestinal effects of digestion-derived fractions remain insufficiently characterized. The present research combined INFOGEST 2.0 in vitro digestion with a polarized Caco-2 monolayer model to examine the potential intestinal effects of digestates produced from *Tenebrio molitor* and *Alphitobius diaperinus*. Neither digestate affected cell viability at the tested concentrations. Transepithelial electrical resistance also remained comparable to the control treatments, indicating the preservation of monolayer integrity. Nevertheless, both insect-derived digestates moderately increased Lucifer Yellow apparent permeability. At the molecular level, both treatments significantly upregulated genes associated with epithelial differentiation and tight junctions, including claudins and intestinal alkaline phosphatase, while *A. diaperinus* digestate additionally increased the mRNA expression of tight junction protein 1 and sucrase-isomaltase. Both digestates also enhanced the expression of antioxidant-related genes, including NAD(P)H quinone dehydrogenase 1, glutathione-disulfide reductase, and heme oxygenase 1, with *A. diaperinus* eliciting a broader transcriptional response than *T. molitor* by additionally increasing the expression of NFE2-like bZIP transcription factor 2 and superoxide dismutase 1. Overall, these results indicate that digestion-derived components from these edible insects can modulate selected intestinal epithelial and antioxidant-related transcriptional responses in differentiated Caco-2 cells under the conditions tested.

## Simple Summary

The growing demand for sustainable protein sources has increased interest in edible insects for feed and food applications. However, less is known about how compounds released during their digestion may interact with intestinal epithelial cells. To fill this knowledge gap, the current research investigated the effects of *Alphitobius diaperinus* and *Tenebrio molitor* digestates on Caco-2 intestinal epithelial cells, focusing on the expression of genes related to epithelial barrier function and oxidative stress. The results showed that treatments with the insect-derived digestates maintained or increased the expression of genes associated with intestinal cell integrity, together with changes in antioxidant defense-associated gene expression. These findings indicate the potential of edible insect-derived digestion products to modulate intestinal epithelial responses in the Caco-2 cell model. However, the present in vitro findings do not establish beneficial effects on intestinal health in animals or humans, and further studies are required.

## 1. Introduction

Numerous global challenges, such as the rapid growth of the population, climate change, and various public health issues have affected food and feed production systems [1,2,3,4]. The demand for alternative protein sources that can meet future nutritional demands while reducing the environmental impact of conventional livestock rearing has been driven from the transition toward more sustainable food systems [5,6]. Among the emerging options, edible insects have gained increasing scientific and industrial interest [7,8,9,10], as they can be produced with relatively low requirements for water, feed, and land, while producing fewer emissions of greenhouse gases [11,12,13].

Beyond their potential as alternative protein sources for human consumption, edible insects have attracted considerable attention as feed ingredients for livestock, poultry, and aquaculture [14]. Their high protein content, favorable amino acid profile, and other valuable nutrients support their incorporation into animal diets and their use as partial substitutes for conventional protein sources, including fishmeal and soybean meal [15]. Their efficient feed conversion, together with their ability to bioconvert low-value agro-food by-products into protein-rich biomass, further enhances their potential for use in animal production [16].

Many edible insect species possess a favorable nutritional profile characterized by essential fatty acids, high-quality proteins, minerals, vitamins, and other bioactive constituents [17,18,19], supporting their incorporation into food and feed as nutritionally valuable ingredients. In particular, edible insects have emerged as prospective bioactive peptide sources, with insect protein hydrolysates reported to display a variety of biological processes, such as antihypertensive, anticancer, antimicrobial, anti-inflammatory, antioxidant, and immunomodulatory effects [20]. Among the edible insects, the coleopterans *Alphitobius diaperinus* (Panzer) and *Tenebrio molitor* L. (Tenebrionidae) are two of the most commercially important species [21,22]. However, the presence of biological activity in protein hydrolysates does not necessarily translate into physiological effects in vivo, as gastrointestinal digestion and intestinal transport may influence peptide bioaccessibility, bioavailability, and biological activity [23,24]. Accordingly, combining physiologically relevant in vitro gastrointestinal digestion using the INFOGEST protocol with subsequent evaluation in an intestinal epithelial Caco-2 cell model offers a more comprehensive approach to investigate the intestinal transport and potential biological effects of digestion-derived peptides [25].

The intestinal epithelium constitutes a dynamic and selectively permeable barrier that regulates the transepithelial passage of nutrients and other luminal compounds while restricting the uncontrolled translocation of potentially harmful substances into the underlying tissues [26]. Upon differentiation, Caco-2 cells develop polarized, enterocyte-like monolayers characterized by tight junction formation, an apical brush border structure, and the expression of intestinal digestive enzymes, such as sucrase-isomaltase (SI) and alkaline phosphatase (ALPI), supporting their use as an established in vitro model for investigating intestinal epithelial barrier function and transepithelial transport [25,27,28].

The intercellular space between adjacent intestinal epithelial cells is tightly regulated by a network of junctional complexes, including desmosomes, adherence junctions, and tight junctions (TJs) [29]. TJs are specialized multiprotein complexes located at the apical region of the lateral membrane of intestinal epithelial cells, where they regulate paracellular permeability and contribute to the maintenance of epithelial barrier integrity [29]. Their structure comprises transmembrane proteins, including claudins (CLDNs) and occludin (OCLN), together with cytoplasmic scaffolding proteins such as zonula occludens-1 (ZO-1), which link junctional complexes to the intracellular actin cytoskeleton [30,31]. Alterations in the expression or organization of these proteins can consequently compromise epithelial barrier function. In light of this, the barrier properties and transepithelial transport characteristics of differentiated Caco-2 monolayers can be evaluated using complementary approaches, including apparent permeability (P_app_) and transepithelial electrical resistance (TEER), which provide functional measures of epithelial barrier integrity and compound permeability, respectively [32]. At the molecular level, changes in epithelial barrier properties can be further investigated by assessing the expression of genes encoding TJ-associated proteins, including *OCLN*, *TJP1*, and members of the *CLDN* family.

Beyond their barrier function, intestinal epithelial cells are actively involved in maintaining cellular redox homeostasis through strongly regulated antioxidant defense mechanisms. The intestinal epithelium is continuously exposed to reactive oxygen species (ROS) and other oxidative challenges (e.g., hydrogen peroxide, lipid hydroperoxides, and reactive nitrogen species) arising from both physiological metabolic processes and luminal factors, requiring coordinated redox regulation to preserve epithelial homeostasis and cellular integrity [33,34]. The nuclear factor erythroid 2-related factor 2 (Nrf-2), encoded by the NFE2-like BZIP transcription factor 2 (*NFE2L2*) gene, represents a central regulator of the cellular antioxidant and cytoprotective response [35,36]. Under basal conditions, Nrf-2 activity is tightly regulated, whereas oxidative or electrophilic stress promotes its stabilization and nuclear translocation, leading to the transcription of antioxidant and cytoprotective genes, including superoxide dismutase 1 (*SOD1*), catalase (*CAT*), heme oxygenase 1 (*HMOX1*), glutathione-disulfide reductase (*GSR*), and NAD(P)H quinone dehydrogenase 1 (*NQO1*) [37].

Although there is an escalating interest in the biological properties of *A. diaperinus* and *T. molitor*, studies specifically investigating the effects of their gastrointestinal digestion-derived constituents on intestinal epithelial function remain limited, particularly regarding their potential effects on intestinal barrier function and cellular antioxidant responses [38]. In a recent study, digestion-derived fractions from both species exhibited antioxidant activity in cell-free assays and induced changes in the expression of genes involved in antioxidant defense under oxidative stress conditions [39]. These findings prompted further investigation of whether the biological activity of insect digestates could also extend to intestinal epithelial cells. Based on the reported biological activities of insect-derived digestion products, we hypothesized that digestates obtained from *A. diaperinus* and *T. molitor* larvae could modulate intestinal epithelial responses in differentiated Caco-2 cells, as reflected by changes in monolayer integrity, paracellular permeability, and the expression of genes associated with epithelial differentiation, tight junctions, and cellular antioxidant responses. Accordingly, this study investigates, for the first time, the effects of whole intestinal digestates from *A. diaperinus* and *T. molitor* on differentiated Caco-2 monolayers, focusing on epithelial barrier function and permeability, as well as on the gene expression of molecular markers associated with epithelial differentiation, TJs, and antioxidant defense.

## 2. Materials and Methods

### 2.1. Tenebrio molitor and Alphitobius diaperinus Colonies and Preparation

Larvae of both species (*T. molitor* and *A. diaperinus*) were obtained from colonies of the Laboratory of Agricultural Zoology and Entomology (Athens, Greece). Both insects were reared under constant abiotic conditions: 65% relative humidity, 30 °C, and under continuous darkness [40,41,42]. Different larval colonies of *Alphitobius diaperinus* were maintained on a substrate consisting of 75% wheat bran and 25% yeast, with apple slices supplied as a moisture source [43]. *Tenebrio molitor* larvae, maintained at different colonies, were reared on oat bran supplemented with potato pieces to provide additional moisture [44]. Larvae selected for the experiments exceeded 7 mm in length in the case of *T. molitor*, whereas *A. diaperinus* larvae measured between 10 and 14 mm [39]. For each insect species, 2 × 55 g of fresh larvae, originating from different colonies, were collected. Following separation from the rearing substrate, larvae were deprived of feed for 24 h to allow for the evacuation of gut contents [45,46,47]. After fasting, any dead individuals were discarded by visual examination, and the remaining larvae were washed three consecutive times with distilled water. The larvae were subsequently inactivated by blanching in hot water (1–5 min), followed by cooling and removal of the surface water. Batches were blanched at 88 °C for 90 s in the case of *T. molitor* and at 93 °C for 150 s for *A. diaperinus*. The processed larvae were frozen at −20 °C until further analyses. Two larval pools (50 g each) were finally collected for each species from two different rearing batches. Each biological batch was processed separately through freeze-drying, grinding, and subsequent sample preparation (described in Section 2.2.), resulting in two independent samples for each insect species. From each biological batch, two separate samples were prepared and subjected independently to the digestion procedure, resulting in four digestion samples per insect species (two biological batches × two digestion replicates per batch). Technical replicates were subsequently used for the cell-based analyses, with the number of replicates specified in the respective sections below.

### 2.2. In Vitro Gastrointestinal Digestion Using the Protocol INFOGEST

The protein content of *A. diaperinus* and *T. molitor* larvae was determined by the Kjeldahl method according to standard AOAC procedures [48], using an appropriate nitrogen-to-protein conversion factor for each insect species [49]. Protein determination was performed in two independent rearing batches, with duplicate analytical measurements for each batch, and the resulting protein concentrations were used to normalize the amount of insect biomass subjected to in vitro gastrointestinal digestion. Both larval biomasses were freeze-dried under controlled conditions, with the shelf temperature gradually increased from −20 °C to 15 °C in 5 °C steps at 4 h intervals. During the process, the temperature difference between the shelf and the samples was maintained below 10 °C, while the chamber pressure was kept at 1 mbar. Following lyophilization, the dried insect material was reconstituted in 5 mL of Milli-Q water (Merck Millipore, Burlington, MA, USA) and allowed to rehydrate overnight at 4 °C to facilitate complete dispersion and solubilization.

The rehydrated samples were subsequently subjected to an in vitro gastrointestinal digestion procedure based on the INFOGEST 2.0 standardized protocol [50], with minor adaptations. For each insect species, two separate digestion experiments were performed for each biological batch, resulting in four digestion experiments per species. The samples were adjusted to an equivalent protein input of 0.5 g per digestion. The digestion process comprised sequential oral, gastric, and intestinal phases. The oral step was conducted at 37 °C for 2 min in the presence of 0.17 mL of α-amylase (E-BLAAM enzyme activity 3000 U/mL; Megazyme, Wicklow, Ireland) [51], followed by the gastric and intestinal stages as previously described by Dalaka et al. [52]. At the completion of the intestinal phase (protein concentration of 0.0625 g/mL), the digestates (*A. d.*-D for *A. diaperinus* larvae and *T. m.*-D for *T. molitor* larvae) were heated at 85 °C for 10 min, cooled on ice, and centrifuged at 1200× *g* for 5 min. Then, the resulting supernatants were passed through 0.22 μm sterile PVDF syringe filters (Merck Millipore, Burlington, MA, USA) and remained at −20 °C until further analyses. In parallel, four independent blank digestions (BL-D) were performed under identical conditions by replacing the insect material with Milli-Q water.

### 2.3. Caco-2 Cell Culture, Differentiation, Cell Viability, and Monolayer Integrity

Caco-2 cells were cultured in supplemented DMEM medium (Biosera, Cholet, France) containing 10% (*v*/*v*) FBS (Gibco ThermoFisher Scientific, Waltham, MA, USA), 1% (*v*/*v*) penicillin-streptomycin antibiotics (Biosera, Cholet, France), 1% (*v*/*v*) non-essential amino acids (Biosera, Cholet, France), and 1% (*v*/*v*) sodium pyruvate (Biosera, Cholet, France). Cells were incubated at 37 °C in a humidified incubator containing 5% CO_2_. Caco-2 cells were seeded at 0.5 × 10^6^ cells/mL in a 25 cm^2^ tissue culture flask (SPL Life Sciences, Pocheon, Republic of Korea) and detached with 0.05% trypsin (Pan-Biotech, Aidenbach, Germany) every 2 or 3 days prior to reaching confluence. The passage number of the cells used in this study was between 10 and 15 (passage was defined as 1 when they arrived in the laboratory).

Caco-2 cells were seeded into standard 12-well cell culture plates containing PET SPLInsert™ hanging inserts (10.5 mm diameter, 0.4 μm membrane pore size, 0.9 cm^2^ growth area, SPL Life Sciences, Pocheon, Republic of Korea) at an initial concentration of 5 × 10^5^ cells/mL [53]. Cells were cultured for 21 days, with the culture medium replaced every 2–3 days, following the protocol proposed by Hubatsch et al. [53]. Cell differentiation was confirmed by measuring TEER, which reached approximately 800 Ω·cm^2^ at the end of differentiation, using a Millicell ERS-2 Digital Voltohmmeter (Merck Millipore, Burlington, MA, USA), together with the microscopic observation of dome formation.

Briefly, TEER was monitored throughout the differentiation period (days 0, 4, 6, 9, 13, 15, 17, 19, and 21) to assess the progressive establishment and integrity of the Caco-2 monolayers. Measurements were performed using a Voltohmmeter equipped with chopstick electrodes. Prior to each measurement, the electrodes were sterilized with isopropanol and equilibrated in phosphate-buffered saline (PBS; Takara Bio, Shiga, Japan). Measurements were conducted within 5 min after removing the plates from the incubator to minimize temperature-related variations. TEER values were normalized to the effective membrane area and expressed as Ω·cm^2^. Cell-free inserts were used as controls, and the corresponding background resistance was subtracted from the measurements obtained from the cell-containing inserts [54].

On day 21 of differentiation, TEER measurements were continued throughout the treatment period to monitor monolayer integrity and ensure that the experimental treatments did not compromise epithelial barrier function [25]. An initial TEER measurement (0 h) was recorded immediately before treatment. Subsequently, 0.5 mL of supplemented DMEM containing the respective digested treatments (*A. d*.-D, *T. m.*-D, or BL-D) was added to the apical compartment of the inserts, while 1.5 mL of DMEM was added to the basolateral compartment. Then, the cells were incubated at 37 °C, and TEER was recorded again after 2 h and at the end of the treatment period (4 h). Inserts without cells were used as controls. The background resistance was subtracted from each measurement, and TEER values were expressed as Ω·cm^2^ after normalization to the effective membrane area (0.9 cm^2^).

Prior to the main experiments, the 3-(4,5-dimethylthiazol-2-yl)-2,5-diphenyltetrazolium bromide (MTT) assay was performed to determine the optimal dilution of the samples, defined as the highest concentration that did not induce cytotoxicity. Caco-2 cells were seeded in a 96-well plate at a density of 5 × 10^4^ cells/well and cultured until reaching confluency [55]. The culture medium was subsequently replaced with new medium containing different concentrations of the digested insect samples, corresponding to 2.5%, 5%, and 10% (*v*/*v*), equivalent to 1562, 3125, and 6250 μg protein/mL, respectively. Cells were incubated with the samples for 4 h to approximate a short-term intestinal exposure period, as previously described in comparable in vitro digestion/Caco-2 cell studies [56,57]. Following incubation, the supernatant was removed, and 0.5 mg/mL MTT solution (Duchefa Biochemie BV, Haarlem, The Netherlands) was added and incubated for an additional 3 h to allow formazan crystal formation. The supernatant was then discarded, and the resulting formazan crystals were dissolved in 2-propanol. Using a TECAN Infinite M200 microplate reader (Tecan Life Sciences, Männedorf, Switzerland) the absorbance was measured at 570 nm. For each sample, three technical replicate wells were used for cell treatment, and cell viability was expressed as a percentage relative to the control cells, which were treated with DMEM alone.

### 2.4. Assessment of Paracellular Permeability Using Lucifer Yellow

Following the 4 h treatment period, cells were washed with HBSS (Biosera, Cholet, France) containing 5 mM HEPES (Biosera, Cholet, France), and plates were equilibrated at 37 °C for 15 min [53,58]. For the assessment of apparent paracellular permeability, 0.4 mL of Lucifer Yellow (LY) solution (100 μM in HBSS containing 5 mM HEPES, pH 7.4) was added to the apical compartment, while 1.2 mL of the same transport buffer was added to the basolateral compartment. The inserts were incubated at 37 °C for 30 min. At the end of the incubation period, an aliquot was collected from the basolateral compartment and transferred to a black, clear-bottom 96-well microplate (Gibco ThermoFisher Scientific, Waltham, MA, USA). LY fluorescence was measured using a FLUOstar Omega microplate (BMG Labtech, Ortenberg, Germany) reader at an excitation wavelength of 485 nm and an emission wavelength of 520 nm. The apparent permeability coefficient (P_app_) of LY across the Caco-2 monolayers was calculated according to Equation (1):

(1) $$Papp=\frac{C_{BL}\times V_{BL}}{C_{0}\times A\times t}$$

where C_BL_ is the LY concentration in the basolateral compartment calculated from the calibration curve (μM), V_BL_ is the basolateral compartment volume (mL), C_0_ is the initial LY concentration (μM) in the apical compartment, A is the membrane area (cm^2^), and t is the incubation time (s). LY concentrations in the basolateral samples were determined from the corresponding fluorescence values using the calibration curve. Measurements of each treatment were performed in quadruplicate.

### 2.5. Gene Expression Analysis of Tight Junction, Differentiation, and Antioxidant Related-Genes

Following the respective treatments, the total RNA from inserts was isolated using Nucleozol reagent (Macherey-Nagel, Düren, Germany). Genomic DNA was removed by DNase treatment (New England Biolabs, Ipswich, MA, USA). Subsequently, cDNA was synthesized by using the PrimeScript RT Reagent Kit (Takara Bio, Kusatsu, Shiga, Japan) and qPCR was performed using FastGene IC Green 2X qPCR Universal Mix (Nippon Genetics, Düren, Germany) following the manufacturers’ instructions. Each sample was analyzed in duplicate as technical replicates, and the mean Ct value of the two technical replicates was used for subsequent relative gene expression analysis. Primers were designed in-house with an annealing temperature at 60 °C. Relative gene expression levels were normalized to the housekeeping genes (*HPRT1* and *GAPDH*) [59,60,61]. Primer details are provided in Table 1.

### 2.6. Statistical Analysis

GraphPad Prism 8 (GraphPad Software, San Diego, CA, USA) was used for the graphical representations, and SPSS software (version 22.0.0; IBM Corp., Armonk, NY, USA) was used for the statistical analyses. The results are presented as the mean ± standard error of means (SEMs) for MTT, TEER, and gene expression analysis, whereas P_app_ values are presented as mean ± standard deviation (SD). The distribution of the data was evaluated by using the Kolmogorov–Smirnov test, and homogeneity of variances was assessed using Levene’s test. For gene expression analysis, where appropriate, data were log-transformed (*ALPI* and *SI*) or normalized (*OCLN*, *TJP1*, and *SOD1*) prior to statistical analysis to improve conformity with the assumptions of the statistical tests [62]. Statistical conclusions were based on the transformed or normalized datasets, while the figures are presented on the original scale to facilitate biological interpretation. For each insect species, four digestion experiments were included in the statistical analysis, corresponding to two separate digestions performed from each of two biological batches. The digestion replicate considered the experimental unit for all statistical analyses, and the four digestion-derived samples per insect species were treated as independent experimental observations. Technical replicates from the subsequent cell-based experiments and qPCR analyses were averaged prior to statistical analysis and were not considered independent observations. One-way ANOVA (analysis of variance) followed by Tukey’s post hoc test was used to assess differences among treatments. Effect sizes were additionally estimated using eta squared (η^2^). Given the number of genes evaluated and the multiple statistical comparisons performed, the gene expression analyses were considered exploratory, and the potential for inflated type I error rates associated with multiple testing is acknowledged. Statistical significance was determined at the 95% level of confidence (*p* ≤ 0.05).

## 3. Results

### 3.1. Validation of the Caco-2 Model and Assessment of Cell Viability, Monolayer Integrity, and Paracellular Permeability

The cytotoxic potential of the insect digestates was initially evaluated using the MTT assay in undifferentiated Caco-2 cells to determine the concentration to be used in subsequent experiments with differentiated Caco-2 monolayers. Undifferentiated Caco-2 cells were exposed to three concentrations (2.5%, 5%, and 10% *v*/*v*) of *A. diaperinus* and *T. molitor* digestates for 4 h (Figure 1). Cell viability was not significantly affected by any of the tested concentrations compared with the DMEM-treated control cells (*p* > 0.05). Similarly, no significant differences in cell viability were observed among the tested concentrations within either insect digestate. Based on these findings, the highest concentration tested (10% *v*/*v*) was selected for the following experiments using differentiated Caco-2 monolayers. This concentration did not compromise cell viability while enabling the assessment of the biological effects of the insect digestates.

The integrity of the differentiated Caco-2 monolayers was first validated by TEER measurements, which reached values of approximately 800 Ω·cm^2^ by day 21 of culture, confirming the establishment of functionally intact epithelial monolayers suitable for subsequent experiments (Supplementary Figure S1). During the experimental period, TEER was monitored to assess monolayer integrity and determine whether the applied treatments affected epithelial barrier function. As shown in Figure 2, TEER values of the DMEM-treated control monolayers increased from approximately 960 Ω·cm^2^ at the beginning of the experiment to 1140 Ω·cm^2^ at 120 min, followed by a slight decrease to approximately 1080 Ω·cm^2^ at 240 min. The BL-D inserts showed relatively stable TEER values, ranging from approximately 960 to 985 Ω·cm^2^ throughout the experimental period. Treatment with *A. d.*-D and *T. m.*-D resulted in TEER profiles comparable to those of the DMEM-treated control monolayers, with no statistically significant differences observed throughout the experimental period (*p* > 0.05). Overall, the maintenance of relatively high TEER, together with the absence of statistically significant differences between treatments and the control, indicates that neither insect-derived digestate markedly compromised Caco-2 monolayer integrity under the experimental conditions.

To further evaluate the effect of the insect-derived digestates on epithelial barrier permeability, the P_app_ of LY was determined across the differentiated Caco-2 monolayers (Table 2). The P_app_ values obtained for the HBSS control and BL-D were comparable, with no statistically significant difference between these groups (*p* > 0.05). In contrast, treatment with *A. d.*-D and *T. m.*-D resulted in significantly higher P_app_ values, compared with both control groups (*p* < 0.05). No significant difference was observed between the two insect-derived digestates (*p* > 0.05). These findings indicate that both insect digestates moderately increased the paracellular permeability of the Caco-2 monolayers, as reflected by the higher LY P_app_ values, despite the absence of significant alterations in TEER during the treatment period.

### 3.2. Effects of Insect Digestates on the Expression of Differentiation and Tight Junction-Related Genes

To determine whether insect-derived digestates affect intestinal epithelial differentiation and barrier-related gene expression, the mRNA levels of *CLDN2*, *CLDN12*, *CLDN15*, *OCLN*, *ALPI*, *TJP1*, and *SI* were evaluated in differentiated Caco-2 cells (Figure 3). Incubation of Caco-2 with either *A. d.*-D or *T. m.*-D led to a significant upregulation in the expression levels of the *CLDN2* (*p* < 0.001; η^2^ = 0.550), *CLDN12* (*p* = 0.011; η^2^ = 0.301), and *CLDN15* (*p* = 0.034; η^2^ = 0.209) genes compared to the cells treated with BL-D (Figure 3a–c). A similar pattern was observed for the functional enterocyte marker *ALPI*, with both treatments significantly increasing (*p* = 0.001; η^2^ = 0.391) its expression compared with BL-D (Figure 3e). In contrast, the *OCLN* (*p* < 0.001; η^2^ = 0.441), *TJP1* (*p* < 0.001; η^2^ = 0.634) and *SI* (*p* = 0.001; η^2^ = 0.363) mRNA levels were significantly higher following treatment with *A. d.*-D compared with both BL-D and *T. m.*-D, whereas no significant differences were observed between *T. m.*-D and BL-D (Figure 3d, Figure 3f, and Figure 3g, respectively). Overall, these findings demonstrate that insect-derived digestates modulated the expression of genes associated with epithelial differentiation and TJ formation, with some of the observed effects being greater in magnitude following treatment with the *A. diaperinus*-derived digestate.

### 3.3. Effects of Insect Digestates on the Expression of Antioxidant-Related Genes

To further elucidate the biological response of differentiated Caco-2 cells following treatment with *A. diaperinus*- and *T. molitor*-derived digestates, the expression of key genes involved in the antioxidant response, including *NFE2L2*, *HMOX1*, *SOD1*, *CAT*, *GSR*, and *NQO1*, was evaluated by qPCR. The results are depicted in Figure 4a–f. Treatment with *A. d.*-D significantly increased the *NFE2L2* (*p* = 0.007; η^2^ = 0.283) and *SOD1* (*p* < 0.001; η^2^ = 0.613) mRNA levels compared with BL-D as well as *T. m.*-D (Figure 4a and Figure 4c, respectively). Both insect-derived digestates significantly upregulated *HMOX1* (*p* < 0.001; η^2^ = 0.524), *GSR* (*p* < 0.001; η^2^ = 0.492), and *NQO1* (*p* < 0.001; η^2^ = 0.528) expression compared with BL-D (Figure 4b, Figure 4e and Figure 4f, respectively). No significant changes (*p* > 0.05; η^2^ = 0.015) were observed for *CAT* expression following treatment with either insect-derived digestate (Figure 4d). Overall, the *A. diaperinus*-derived digestate elicited a broader induction of antioxidant-related transcriptional response compared with the *T. molitor*-derived digestate.

## 4. Discussion

According to the results of the current study, the crude protein content of larvae was 42.17 ± 1.49% for *A. diaperinus* and 44.05 ± 2.12% for *T. molitor*. These values are within the range of protein contents formerly reported for both species [63,64,65], although differences among studies may arise from variations in diet, analytical methodology, rearing conditions, and developmental stage [66]. The protein content was used to standardize the digestion input, with 0.5 g of protein used for each digestion, ensuring comparable protein inputs between the two insect species [67,68]. However, the nutritional value of insect proteins alone does not fully capture their potential as functional ingredients, as gastrointestinal digestion generates peptides, fatty acids, and other digestion-derived compounds that may interact with intestinal epithelial cells and influence cellular signaling pathways [69]. Importantly, the biological relevance of such in vitro activities also depends on whether digestion-derived compounds remain available at the intestinal epithelium and interact with intestinal epithelial cells [70]. Thus, evaluating insect proteins at the post-digestion level, rather than solely on the basis of their initial protein content, allows for a better understanding of their potential functional effects. Accordingly, the resulting digestates were evaluated for their effects on intestinal barrier integrity and the expression of genes associated with epithelial differentiation, tight junctions, and cellular antioxidant responses using a differentiated Caco-2 monolayer model.

Caco-2 cell viability was assessed, as a first step, following exposure to the *A. diaperinus*- and *T. molitor*-derived digestates. None of the tested concentrations adversely affected cell viability, which remained within the range of approximately 100–130%. Based on these results, the highest tested concentration, corresponding to 10% (*v*/*v*) or 6.25 mg protein/mL, was selected for the subsequent experiments. This concentration represented the highest non-cytotoxic tested concentration under the experimental conditions and was also within the range previously investigated for digestion-derived insect protein fractions in cell-based models. In particular, concentrations of up to 6.25 mg protein/mL have been employed to evaluate < 3 kDa digestion-derived fractions from *A. diaperinus* and *T. molitor* in LPS-stimulated, PMA-differentiated THP-1 macrophages [39]. In contrast to the present findings, Ma et al. [38] reported cytotoxic effects of the <10 kDa fraction of *A. diaperinus* intestinal digestate at concentrations ranging from 2 to 5 mg protein/mL in Caco-2 cells, whereas lower concentrations did not adversely affect cell viability. In a previous study, *T. molitor* hydrolysate did not exert cytotoxic effects on RAW 264.7 macrophages at concentrations of up to 0.5 mg protein/mL [71]. More recently, Lee et al. [72] reported that digested insect protein filtrates were non-toxic to Vero cells, with the <3 kDa fraction derived from *T. molitor* even promoting cell growth. Similarly, Zielińska et al. [73] found that *T. molitor* and one other tenebrionid, *Zophobas atratus* (F.) [=*Zophobas morio* (F.)] (Coleoptera) extracts did not exhibit cytotoxicity toward the mouse endothelial HECa10 cell line at the concentrations of up to 0.1 mg protein/mL. Overall, these findings indicate that several insect-derived protein and digestion-derived fractions exhibit low cytotoxic potential in different cell models under the tested conditions, although the response may vary depending on the insect species, fraction characteristics, concentration, and cellular model. Nevertheless, the selected concentration should be considered an experimental concentration and should not be interpreted as directly representing the concentration of digestion-derived compounds reaching the intestinal epithelium in vivo. Future studies incorporating dose–response designs and physiologically relevant exposure concentrations will be valuable to further elucidate concentration-dependent cellular responses and their potential relevance to intestinal conditions.

As cell viability alone does not provide information on the functional integrity of the intestinal epithelial barrier, TEER and P_app_ were subsequently assessed to further characterize barrier integrity and permeability. Throughout the 21-day differentiation period, TEER values progressively increased, reaching approximately 820 Ω cm^2^ at day 21, immediately prior to 4 h exposure to the digestates. This increase in TEER was consistent with previous studies reporting the development of the Caco-2 epithelial barrier during differentiation [74]. It should be noted, however, that absolute TEER values can vary substantially across studies, reflecting differences in cell passage number, seeding density, culture conditions, membrane properties, and differentiation protocols [75,76,77].

On day 21, TEER values remained unchanged following 4 h exposure to either digestate, whereas both treatments induced a slight but significant increase in epithelial permeability, as reflected by the LY P_app_ values. The absence of significant changes in TEER following exposure to the insect-derived digestates is consistent with previous findings showing that 3 h exposure to corresponding INFOGEST-derived products from the dipteran *Hermetia illucens* (L.) (Stratiomyidae) and *T. molitor* larvae did not markedly impair small intestinal-like Caco-2 barrier integrity [78]. More recently, Barberis et al. [79] reported that an in vitro digested preparation of the orthopteran *Locusta migratoria* (L.) (Acrididae) did not significantly alter TEER or induce morphological changes indicative of epithelial monolayer disruption in Caco-2 cells, further supporting the absence of major barrier-damaging effects of digested insect-derived products. A complementary in vitro study using differentiated Caco-2 cells under LPS-induced inflammatory conditions showed that 24 h exposure to a bioaccessible fraction derived from digested pasta enriched with 10% powder of the orthopteran *Acheta domesticus* (L.) (Gryllidae), diluted 1:30 (*v*/*v*), did not alter TEER [55]. Interestingly, the LY P_app_ values observed in the current study were also within the same order of magnitude as those previously reported for intestinally digested insect-derived fractions, including an *A. diaperinus*-derived fraction, which exhibited a P_app_ value of 0.95 × 10^−6^ cm/s during the 0.5–2 h transport period [38]. Importantly, TEER and LY P_app_ reflect distinct aspects of epithelial barrier integrity and therefore provide complementary information. TEER reflects the electrical resistance of the monolayer, whereas LY P_app_ provides the paracellular passage of a hydrophilic tracer across the monolayer. In the present study, the increase in LY P_app_ in the absence of a significant change in TEER may indicate a subtle increase in paracellular permeability that was not accompanied by a major loss of overall electrical barrier integrity. Accordingly, the two parameters should not be interpreted as contradictory, but rather as a reflection of different aspects and sensitivities of barrier function. Notably, this modest increase in permeability was accompanied by changes in the expression of genes associated with tight junctions and epithelial differentiation, which may reflect a cellular response to digestate exposure. In particular, the increased expression of *CLDN2* may be consistent with the modest increase in paracellular permeability observed by LY P_app_, although *CLDN2* also has physiological roles in epithelial ion and water transport and its increased expression alone does not necessarily indicate barrier disruption [80]. Thus, the observed transcriptional changes may reflect the modulation of barrier-related pathways rather than an unequivocally beneficial effect on barrier function [81].

At the molecular level, both digestates increased the expression of selected genes related to intestinal epithelial differentiation and tight junctions. Neither digestate induced downregulation of the investigated differentiation markers; rather, several genes showed increased expression compared to BL-D. Together with the maintenance of TEER during the 4 h exposure, the absence of downregulation of the investigated differentiation markers suggests that short-term exposure to the digestates did not substantially impair the differentiated enterocyte-like phenotype of the Caco-2 monolayers. This interpretation is consistent with the established use of differentiated Caco-2 monolayers as a model of the enterocyte-like intestinal epithelium, characterized by polarization and the expression of brush-border differentiation markers such as *SI* and *ALPI*, together with tight junction components, including *CLDNs* and *OCLN* [82]. Interestingly, the increased expression observed for several differentiation- and tight junction-related genes suggests that the digestates induced the selective modulation of molecular features associated with the differentiated epithelial phenotype, without clear evidence of impairment. Similar modulation of intestinal barrier-related pathways has been reported following exposure to insect-derived products. In a previous study, Kim et al. [83] showed that the coleopteran *Trypoxylus dichomatus* (L.) [=*Allomyrina dichotoma* (L.)] (Scarabaeidae) larval extract attenuated LPS-induced intestinal barrier disruption in Caco-2 cells, accompanied by changes in the protein levels of ZO-1, occludin, and claudins. Notably, in the absence of LPS stimulation, the larval extract did not induce corresponding changes in the protein levels of these tight junction components. Similarly, the same research group reported comparable findings using an aqueous-ethanolic extract of the orthopteran *Gryllus bimaculatus* De Geer (Gryllidae) [54]. More recently, Teixeira et al. [84] studied the effects of 3 h exposure to peptides obtained from *A. domesticus* digestates on the expression of brush-border peptidases in differentiated Caco-2 cells. Although most peptides did not significantly affect *DPP4* or *sACE* mRNA expression, the peptide DVW significantly reduced *APN* expression, while several peptides underwent extensive hydrolysis by brush-border peptidases. Evidence from other food-derived compounds further supports the selective modulation of tight junction-related genes in differentiated Caco-2 cells. Suzuki and Hara [85] reported that quercetin enhanced the barrier function of Caco-2 monolayers, as evidenced by increased TEER and reduced paracellular LY flux. This effect was associated with enhanced assembly of ZO-2, occludin, and claudin-1 at tight junctions, together with increased claudin-4 expression. Harasawa et al. [86] reported that fructo-oligosaccharides increased *CLDN4* expression while reducing *CLDN2* expression, whereas the expression of *OCLN* and *TJP1* remained unchanged. Similarly, de Simone et al. [87] reported that in vitro digested chickpea-, dark chocolate-, and snack-based products protected Caco-2 monolayers against inflammation-induced barrier disruption, preventing the reduction in TEER induced by LPS and IL-1β. This functional effect was accompanied by the increased expression of tight junction-related genes, particularly *TJP1*, *CLDN1*, and *OCLN* following treatment with the digested chickpea product. Taken together, these findings suggest that insect- and other food-derived digestion products can modulate epithelial and barrier-related pathways without necessarily compromising the differentiated Caco-2 phenotype.

The upregulation of antioxidant-related genes observed in the differentiated Caco-2 model extends previous evidence regarding the antioxidant potential of insect-derived digestion products. Several studies have reported the antioxidant activity of different insect-derived hydrolysates and digestion products in cell-free assays, with processes such as enzymatic hydrolysis, fermentation, and gastrointestinal digestion shown to modulate, and in several cases, enhance their antioxidant capacity [88,89,90,91]. Importantly, this antioxidant potential has also been demonstrated in cell-based models. Lee et al. [72] reported that 1 h treatment with *T. molitor* 3 kDa digestate reduced intracellular ROS production in H_2_O_2_-treated Vero cells. Furthermore, Ma et al. [38] reported a positive correlation between cellular antioxidant activity and intracellular ROS inhibition in Caco-2 cells exposed to gastrointestinal digestates from the lepidopteran *Galleria mellonella* (L.) (Pyralidae) and *A. diaperinus*. Both digestates inhibited intracellular ROS, with the latter showing the highest inhibition (58.5%). Nevertheless, D’Antonio et al. [92] showed that both aqueous extracts and digestates of edible insects reduced free-radical production in AAPH-treated human colonic epithelial cell at all tested concentrations, with the antioxidant effect varying among insect species under in vitro conditions. Likewise, Dutta et al. [93] reported that a hydro-alcoholic extract of the orthopteran *Brachytrupes orientalis* (Burmeister) (Gryllidae) reduced intracellular ROS and lipid peroxidation in high-glucose-treated C2C12 mouse myoblast cells while enhancing the protein expression of the antioxidant defense-related factors Nrf-2 and glutathione S-transferase.

Beyond the reduction in intracellular ROS, a more limited number of studies have investigated the ability of insect-derived hydrolysates to modulate endogenous antioxidant defense mechanisms. Cho and Lee [94] reported that a <1 kDa alcalase hydrolysate of *T. molitor* protected AML12 mouse hepatocytes against H_2_O_2_-induced oxidative stress by reducing intracellular ROS through the Nrf-2-mediated upregulation of antioxidant defense-related genes, including *HMOX1*, *SOD1*, *CAT*, and genes involved in glutathione synthesis. Riolo et al. [95] showed that treatment with *H. illucens* protein hydrolysates attenuated intracellular ROS accumulation in H_2_O_2_-challenged murine L-929 fibroblast cells while also promoting *Nrf2* expression and nuclear translocation. The same research group further reported that *H. illucens* protein hydrolysates upregulated antioxidant-related transcriptional genes, including the transcription factor *Nrf2*, the two *SOD* genes (*SOD1* and *SOD2*), and *HMOX1*, in LPS-challenged L-929 cells. These changes were also supported at the protein level, with treatment significantly increasing the MnSOD and HO-1 levels [96]. More recently, our group [39] demonstrated that the antioxidant-related cellular response to insect-derived digestion products is also dependent on insect species and cellular context. In LPS-stimulated, PMA-differentiated THP-1 macrophages, the <3 kDa intestinal permeate from *A. diaperinus* and *T. molitor* preferentially modulated *NFE2L2*, *HMOX1*, *SOD1*, *CAT*, and *GSR* [39]. In the present study, both digestates modulated antioxidant-related genes, although to different extents, with *A. d.*-D showing a more extensive antioxidant-related transcriptional response. This may reflect differences in experimental design, as previous studies used enzymatic hydrolysates or <3 kDa digestate fractions, oxidative or inflammatory stimuli, and different cellular models, whereas the present study evaluated complete INFOGEST 2.0-derived digestates in differentiated Caco-2 cells under non-challenged conditions. Moreover, the comparison with BL-D rather than untreated cells allowed for insect-specific responses to be distinguished from those associated with the digestion matrix, potentially contributing to the more conservative gene expression differences observed herein.

Further evidence for the antioxidant potential of insect-derived peptides has been provided through the characterization of specific peptide fractions. Summart et al. [97] reported that the most active fraction (F45) obtained from an *A. domesticus* protein hydrolysate enhanced the expression of several antioxidant defense-related genes, including *NFE2L2*, *SOD1*, *SOD2*, *CAT*, and *GSR* in human neuroblastoma SH-SY5Y and human hepatoma HepG2 cell lines. Further fractionation led to the identification of the tetrapeptides, FVEG and FYDQ, which showed potential interactions with antioxidant-related targets, including Keap1, supporting their possible involvement in Nrf-2-mediated antioxidant responses. Simulated gastrointestinal digestion may further modify the antioxidant potential of insect-derived peptide mixtures. De Matos et al. [98] demonstrated that digestion enhanced the antioxidant activity of the orthopteran *Gryllus assimilis* (F.) (Gryllidae) protein hydrolysates, while proteomic analysis identified peptides in the <3 kDa fraction of the digested material, suggesting that digestion-generated peptides may contribute to the observed bioactivity. González-de la Rosa et al. [69] also characterized the peptidome of *T. molitor* hydrolysates and their INFOGEST digestion products, highlighting the generation and persistence of numerous potentially bioactive peptides. These findings support the possibility that the modulation of antioxidant defense-related genes observed in the present study may reflect the combined activity of a complex pool of digestion-derived compounds rather than a single bioactive component. Although peptides may be involved in these responses, the contribution of other digestion-derived constituents, including fatty acids, chitin-derived components, phenolic compounds, and other low-molecular-weight compounds, cannot be excluded. However, as the peptide fractions were not compositionally characterized, future LC-MS/MS-based profiling will be needed to identify the specific peptides potentially contributing to these responses.

Importantly, evidence from in vivo models further supports the potential of insect-derived bioactive compounds to modulate antioxidant defenses. Kim et al. [99] showed that allomyrinasin, a purified peptide derived from *T. dichomatus*, attenuated high-fat diet-induced hepatic oxidative stress and metabolic dysfunction in mice, supporting the potential of insect-derived peptides to modulate endogenous antioxidant defense mechanisms. In vivo evidence has also shown that dietary *T. molitor* can modulate antioxidant defenses, as partial replacement of fishmeal with *T. molitor* meal increased hepatic CAT and GPx activities and the expression of *cat*, *sod*, and *gpx* in juvenile largemouth bass [100]. Similarly, a partial replacement of soybean meal with 25% *T. molitor* meal upregulated two SOD genes (*cu/znsod* and *mnsod)*, *cat*, *nrf2*, and genes involved in glutathione synthesis (*gpx4* and *gr*) and downregulated *keap1* expression in the intestine of grass carp [101], providing further evidence that insect-derived dietary components can influence antioxidant defense pathways at the intestinal level. Also, dietary supplementation with 25% *T. molitor* meal increased *ocln* expression, whereas 100% inclusion levels were associated with reduced *zo-1* and *zo-2* expression. These findings further suggest that the effects of insect-derived dietary components on intestinal barrier-related genes may depend on the inclusion level, with different inclusion levels potentially eliciting distinct molecular responses.

Some limitations should be considered when interpreting the present findings. Τhis study focused on a single in vitro model, specifically differentiated Caco-2 monolayers under non-stressed conditions, exposed to complete INFOGEST 2.0-derived insect digestates. Although this is a well-established and widely used in vitro model to investigate intestinal epithelial barrier properties and cellular responses, it does not fully recapitulate the complexity of the intestinal environment in vivo. Other physiologically relevant intestinal models, including co-culture systems, could therefore provide further insight into the biological significance of the observed effects. In addition, the number of independent biological sources was limited, with two biological batches per insect species and two independent digestion experiments per batch. Therefore, the findings should be considered preliminary and require confirmation in studies including a larger number of independent biological replicates. Τhe exposure period was limited to 4 h, and longer or repeated exposures may result in different molecular and functional responses. Although the combined TEER, LY P_app_, and gene expression data provide complementary evidence of the epithelial responses to the digestates, the modest increase in LY P_app_ indicates that the observed transcriptional responses should not be interpreted as evidence of improved barrier function. The molecular analyses were performed at the mRNA level. Protein-level validation by Western blotting, ELISA, or immunofluorescence would determine whether the observed transcriptional changes are accompanied by corresponding changes in protein expression and/or localization. This is particularly relevant for tight-junction components such as *CLDN2* and *OCLN*, as changes in mRNA expression do not necessarily predict functional barrier effects. More broadly, changes in mRNA abundance do not necessarily translate into corresponding changes in protein expression, localization, or tight-junction assembly. Therefore, the observed upregulation of tight-junction-related genes should be interpreted as a transcriptional response and does not by itself indicate improved tight-junction organization or barrier function. Similarly, the observed changes in antioxidant-related gene expression should not be interpreted as evidence of antioxidant activity per se, as intracellular ROS levels, antioxidant enzyme activities, and protein expression were not measured. Thus, these findings reflect transcriptional regulation of antioxidant defense-related genes rather than direct evidence of antioxidant activity. The inclusion of established positive controls for epithelial barrier modulation or antioxidant signaling could also provide an additional benchmark for model responsiveness and the magnitude of the observed responses. Finally, the complete INFOGEST 2.0 digestates were not subjected to detailed peptide or broader compositional characterization. Therefore, the specific compounds responsible for the observed cellular responses cannot be identified, as the digestates were not subjected to compositional characterization of their major digestion-derived constituents, including peptides, lipids, chitin-derived compounds, and other digestion products. Consequently, no conclusions can currently be drawn regarding which specific components contributed to the observed biological responses or the relative contribution of peptides compared with other constituents of the complete digestates. In particular, peptide profiling and the identification of individual peptide sequences could help determine whether the observed cellular responses are associated with specific digestion-derived peptides or with the collective activity of the digestate. Further studies using characterized digestate fractions, protein-level analyses, time-course experiments, more physiologically relevant intestinal models, and ultimately in vivo approaches will establish the biological and physiological significance of these findings.

## 5. Conclusions

The present study shows that *A. diaperinus* and *T. molitor* digestates resulted in measurable cellular responses in differentiated Caco-2 intestinal epithelial monolayers under the experimental conditions applied. At the transcriptional level, both digestates modulated genes associated with epithelial differentiation and tight junctions, along with changes in the expression of antioxidant defense-associated genes. Although both digestates moderately increased LY P_app_, this effect was not accompanied by a significant reduction in TEER, suggesting that the treatments did not markedly compromise overall monolayer integrity. Overall, the findings provide preliminary evidence that digestion-derived compounds from these edible insects can modulate selected epithelial cellular responses in vitro. Further functional and in vivo studies are required to elucidate the underlying mechanisms, assess the safety and biological significance of these responses, and determine their physiological relevance, particularly in the context of the potential use of these insects as alternative protein sources for feed and food applications.

## Figures and Tables

**Figure 1:**
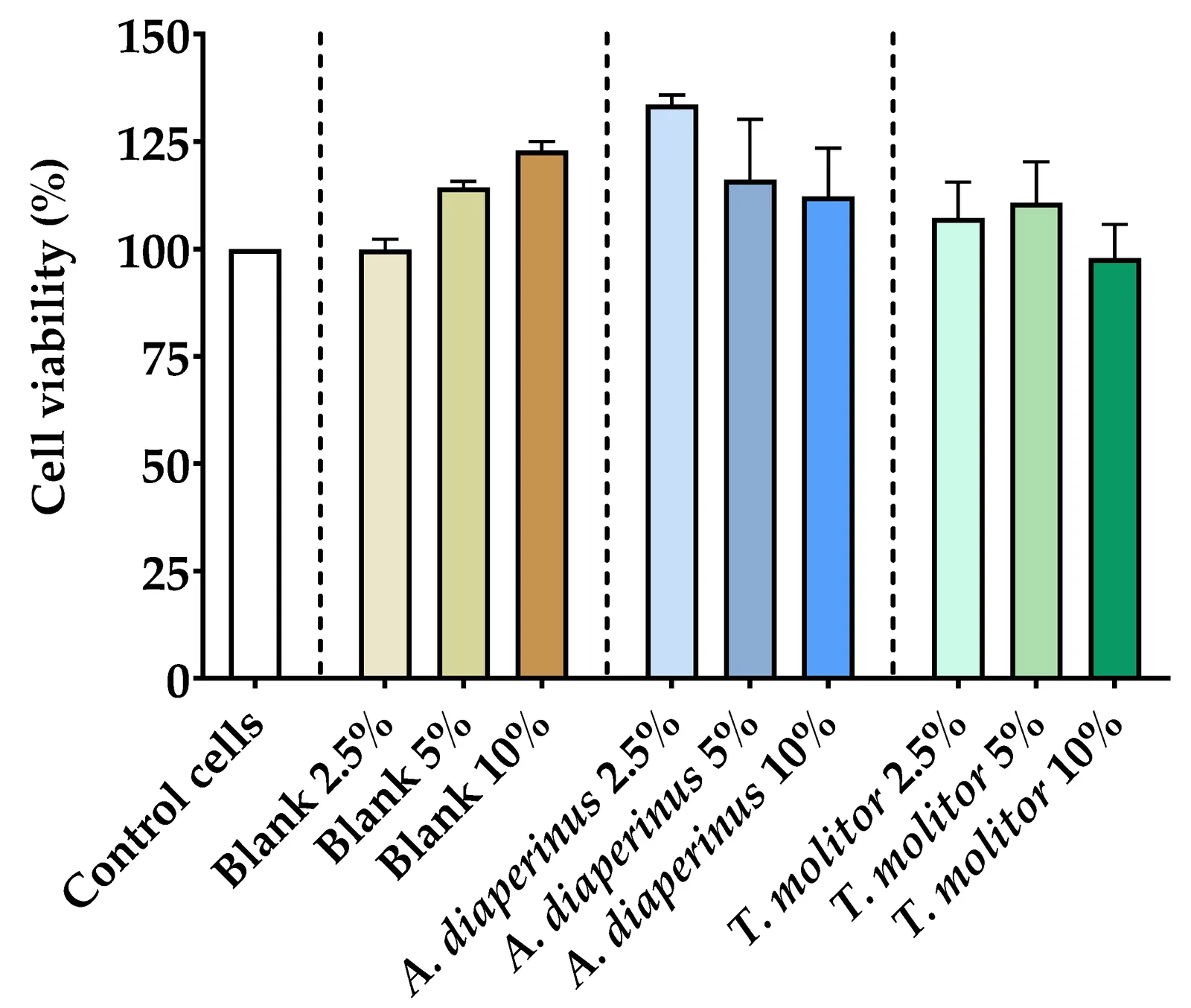
Effects of insect digestates on Caco-2 cell viability. Caco-2 cells were treated for 4 h with *A. diaperinus* digestate (*A. d.*-D), *T. molitor* digestate (*T. m.*-D), and blank digestate (BL-D) at 2.5%, 5%, and 10% (*v*/*v*). DMEM-treated cells were used as the control cells. Data are presented as means ± SEM.

**Figure 2:**
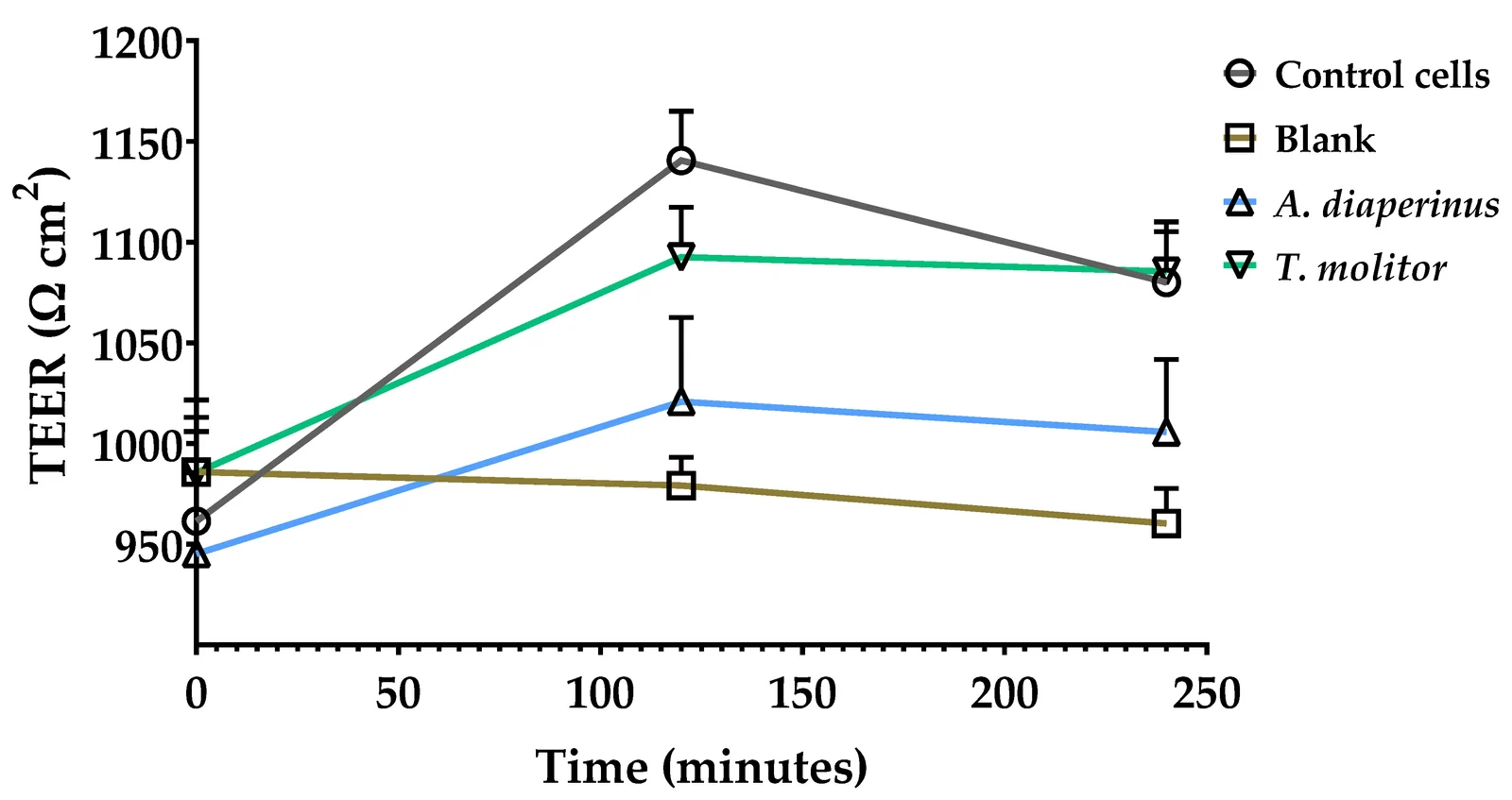
Effects of digestates on the integrity of differentiated Caco-2 cells. Monolayer integrity was assessed by measuring TEER before treatment (0 min), after 120 min, and at the end (240 min) of exposure to *A. diaperinus* digestate (*A. d.*-D), *T. molitor* digestate (*T. m.*-D), blank digestate (BL-D), or DMEM (control cells). Data is presented as means ± SEM. *n* = 3 for DMEM control, *n* = 4 for BL-D, *n* = 2 for *A. d.*-D and *T. m.*-D.

**Figure 3:**
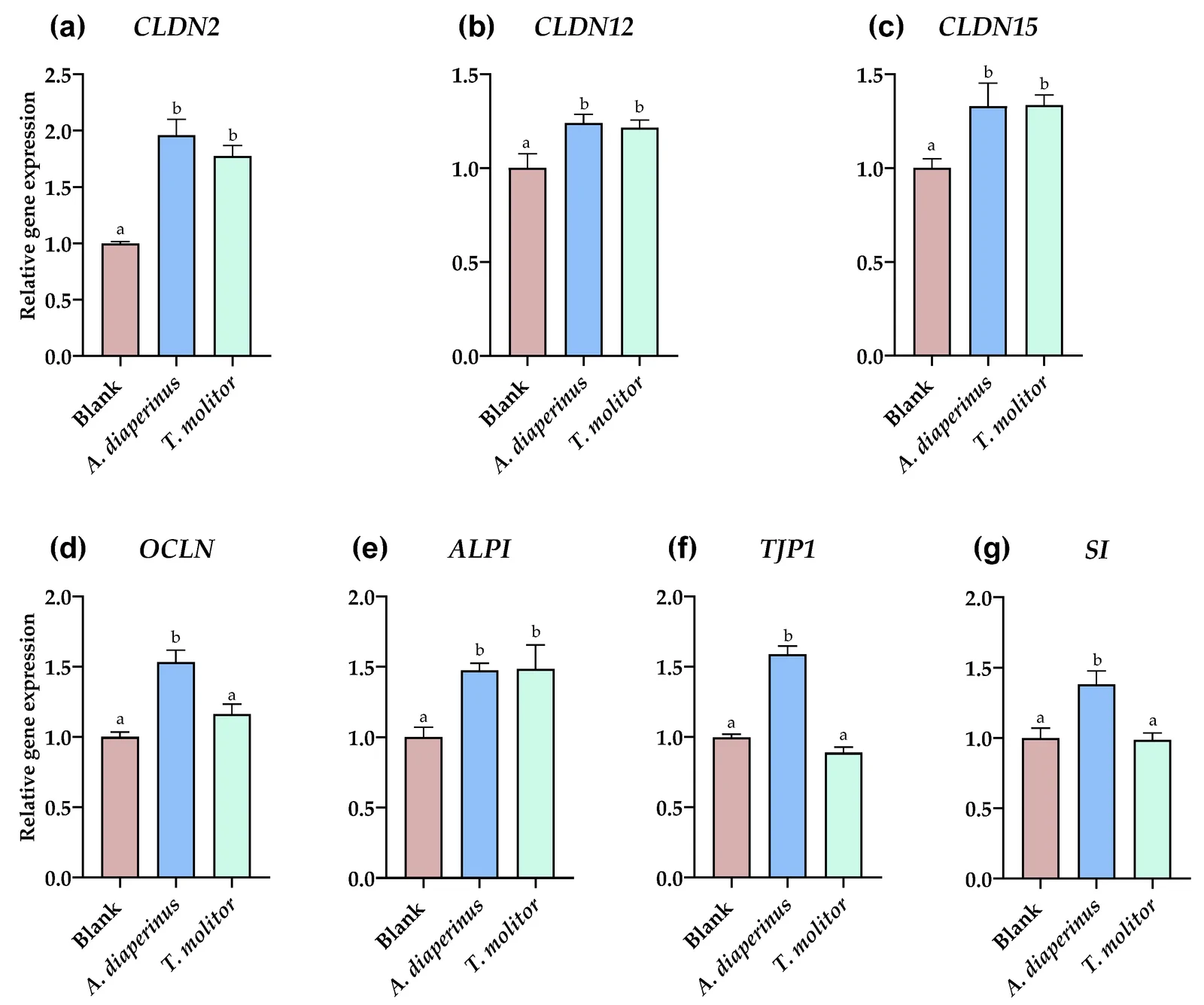
Effects of digestates (*A. d.*-D, *T. m.*-D, and BL-D) on mRNA expression in differentiated Caco-2 cells. Relative mRNA expression of (**a**) *CLDN2*, (**b**) *CLDN12*, (**c**) *CLDN15*, (**d**) *OCLN*, (**e**) *ALPI*, (**f**) *TJP1*, and (**g**) *SI* are presented. Data are presented as means ± SEM. Columns labeled with different letters within the same panel indicate significant differences (*p* ≤ 0.05), as determined by one-way ANOVA followed by Tukey’s post hoc test.

**Figure 4:**
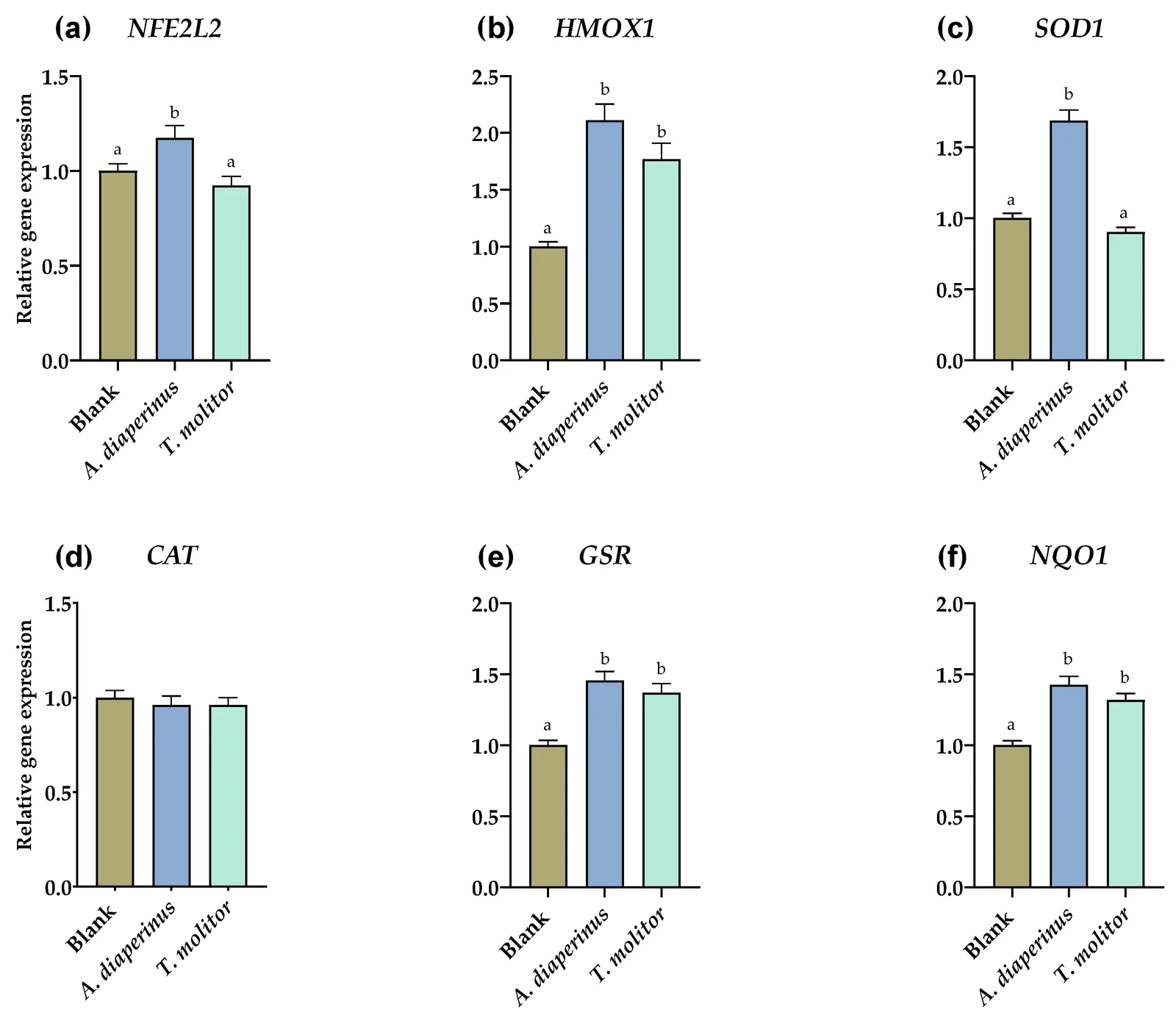
Effects of digestates (*A. d.*-D, *T. m.*-D, and BL-D) on mRNA expression in differentiated Caco-2 cells. Relative mRNA expression of (**a**) *NFE2L2*, (**b**) *HMOX1*, (**c**) *SOD1*, (**d**) *CAT*, (**e**) *GSR*, and (**f**) *NQO1* are presented. Data are presented as means ± SEM. Columns labeled with different letters within the same panel indicate significant differences (*p* < 0.05), as determined by one-way ANOVA followed by Tukey’s post hoc test.

**Table 1:** Oligonucleotide primer sequences, amplicon size, and reaction efficiency in qPCR.

Gene (Accession Number)	Primer Direction	Sequence (5′-3′)	Primer Concentration (nM)	Amplicon Size (bp)	Reaction Efficiency (%)
*CLDN2* (NM_020384.4)	Forward	CGGGACTTCTACTCACCACTG	300	109	110
	Reverse	GGATGATTCCAGCTATCAGGGA			
*CLDN12* (NM_012129.5)	Forward	AGAAGCAGGCTCAGATTATTGC	300	371	98
	Reverse	CTGGTCAACTGATGAGTACCA			
*CLDN15* (NM_014343.3)	Forward	CGGAACCAAGTACGAGCTG	300	221	102
	Reverse	CGTTTCTGCCGTATTTGCC			
*OCLN* (NM_001205255.2)	Forward	ACAAGCGGTTTTATCCAGAGTC	300	89	104
	Reverse	GTCATCCACAGGCGAAGTTAAT			
*ALPI* (NM_001631.5)	Forward	ATGGACATTGACGTGATCCT	300	261	95
	Reverse	TCGGTGGATCTCATATTTCGT			
*TJP1* (NM_001301025.3)	Forward	GAAGAGGATGAAGATGAAGATGG	300	218	107
	Reverse	AGCAGTGTTTCACCTTTCTC			
*SI* (NM_001041.4)	Forward	GCAAATACTAGAAGACAAGATCCC	300	230	104
	Reverse	GTAACCATAAATGCTGGACCC			
*NFE2L2* (NM_006164.5)	Forward	GATCTGCCAACTACTCCCA	200	121	95
	Reverse	GCCGAAGAAACCTCATTGTC			
*HMOX1* (NM_002133.3)	Forward	GCTTCAAGCTGGTGATGG	400	112	100
	Reverse	AGCTCTTCTGGGAAGTAGAC			
*SOD1* (NM_000454.5)	Forward	CGAGCAGAAGGAAAGTAATGG	300	194	103
	Reverse	CCAAGTCTCCAACATGCC			
*CAT* (NM_001752.4)	Forward	TGCCTATCCTGACACTCACC	300	137	102
	Reverse	GAGCACCACCCTGATTGTC			
*GSR* (NM_000637.5)	Forward	CTTGCGTGAATGTTGGATGTG	300	102	91
	Reverse	CACAACTTGGAAAGCCATAATCAG			
*NQO1* (NM_001286137.2)	Forward	AACTTTCAGTATCCTGCCGAG	300	122	99
	Reverse	AGAATGCCACTCTGGAATATCAC			
*HPRT1* (NM_000194.3)	Forward	CTTTGCTTTCCTTGGTCAGG	300	111	99
	Reverse	CAAATCCAACAAAGTCTGGCT			
*GAPDH* (NM_001357943.2)	Forward	CTCATTTCCTGGTATGACAACG	300	185	95
	Reverse	GGGAGATTCAGTGTGGTGG			

**Table 2:** Apparent permeability coefficient (P_app_) of Lucifer Yellow (LY) across Caco-2 monolayers following exposure to insect digestates.

Treatment	P_app_ (×10^−6^ cm/s)
HBSS control	1.69 ± 0.13 ^a^
BL-D	1.71 ± 0.15 ^a^
*A. d.*-D	2.07 ± 0.14 ^b^
*T. m.*-D	2.01 ± 0.12 ^b^

Values are presented as mean ± SD. BL-D, blank digestate; *A. d.*-D, *Alphitobius diaperinus* digestate; *T. m.*-D, *Tenebrio molitor* digestate. Different superscripted letters indicate significant differences between groups (one-way ANOVA followed by Tukey’s test, *p* ≤ 0.05). *n* = 3 for HBSS control, *n* = 4 for BL-D, *n* = 2 for *A. d.*-D and *T. m.*-D.

## Data Availability

The original contributions presented in this study are included in the article. Further inquiries can be directed to the corresponding authors.

## Supplementary Materials

The following supporting information can be downloaded at: https://www.mdpi.com/article/10.3390/biology15191690/s1, Figure S1: Transepithelial electrical resistance (TEER) values of differentiated Caco-2 cell monolayers. Caco-2 cells were seeded onto SPLInsert™ hanging inserts and maintained for 21 days. TEER was measured on days 0, 4, 6, 9, 13, 15, 17, 19, and 21 using a volt-ohm meter equipped with a double chopstick-like electrode. Data are presented as mean ± SEM.

## Abbreviations

The following abbreviations are used in this manuscript:

ALPI	Alkaline phosphatase
AOAC	Association of Official Analytical Chemists
*A. d.*-D	*Alphitobius diaperinus* digestate
BL-D	Blank digestate (digestion with water instead of sample)
*CAT*	Catalase
*CLDN*	Claudin
FBS	Fetal bovine serum
*GAPDH*	Glyceraldehyde-3-phosphate dehydrogenase
*GSR*	Glutathione-disulfide reductase
*HPRT1*	Hypoxanthine phosphoribosyltransferase 1
*HMOX1*	Heme oxygenase 1
K_p_	Nitrogen-to-protein conversion factor
LPS	Lipopolysaccharides from *Escherichia coli* O111:B4
LY	Lucifer Yellow
MTT	3-(4,5-dimethylthiazol-2-yl)-2,5-diphenyltetrazolium bromide
*NFE2L2*	NFE2-like BZIP transcription factor 2
*NQO1*	NAD(P)H quinone dehydrogenase 1
*OCLN*	Occludin
PBS	Phosphate-buffered saline
qRT–PCR	Quantitative reverse transcription polymerase chain reaction
P_app_	Apparent permeability coefficient
ROS	Reactive oxygen species
SI	Sucrase-isomaltase
*SOD1*	Superoxide dismutase 1
TEER	Transepithelial electrical resistance
TJ	Tight junction
*TJP1*	Tight junction protein 1
*T. m.*-D	*Tenebrio molitor* digestate
ZO-1	Zonula occludens-1
