# Supplementary material for: Effects of Alphitobius diaperinus and Tenebrio molitor Larvae Digestates on Intestinal Epithelial Barrier and Antioxidant-Related Gene Expression in Differentiated Caco-2 Cells: An In Vitro Study
Source: Biology (Basel). 2026 Sep 22;15(19):1690. doi: 10.3390/biology15191690 (PMC13663573; doi:10.3390/biology15191690)
Supplement: Supplementary file 1 [file biology-15-01690-s001.zip › biology-4556475-supplementary.pdf]

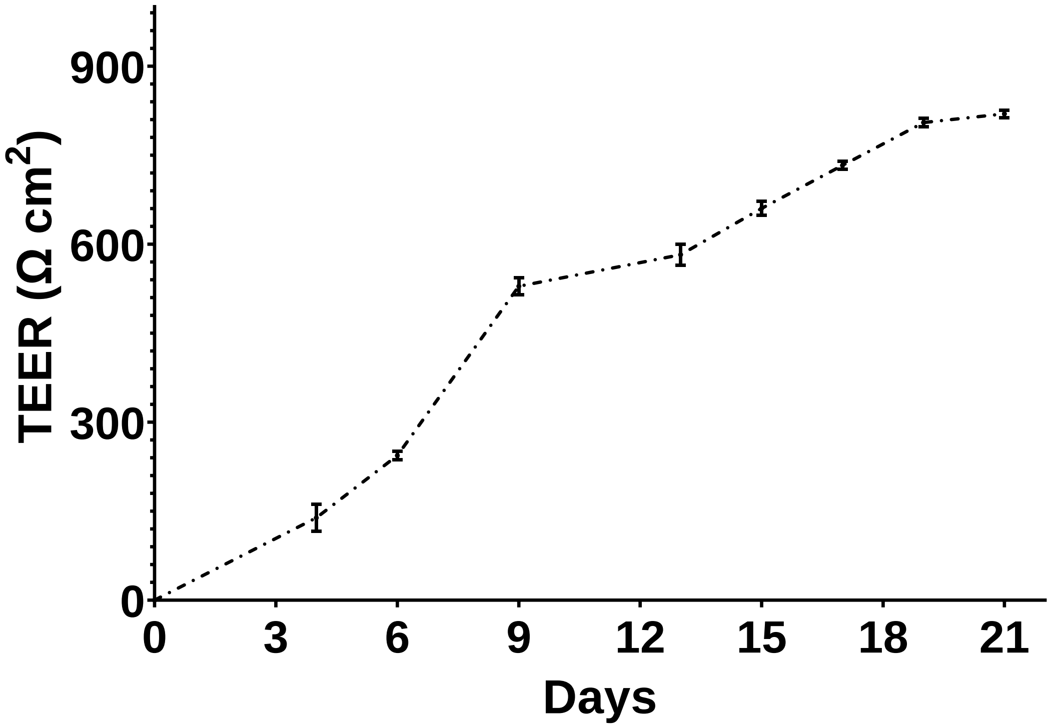

Figure S1. Transepithelial electrical resistance (TEER) values of differentiated Caco-2 cell monolayers. Caco-2 cells were seeded onto SPLInsert™ hanging inserts and maintained for 21 days. TEER was measured on days 0, 4, 6, 9, 13, 15, 17, 19, and 21 using a volt-ohm meter equipped with a double chopstick-like electrode. Data are presented as mean ± SEM.
